# Dissociating implicit and explicit ensemble representations reveals the limits of visual perception and the richness of behavior

**DOI:** 10.1038/s41598-021-83358-y

**Published:** 2021-02-16

**Authors:** Sabrina Hansmann-Roth, Árni Kristjánsson, David Whitney, Andrey Chetverikov

**Affiliations:** 1 Icelandic Vision Lab, School of Health Sciences, University of Iceland, Reykjavík, Iceland; 2 Univ. Lille, CNRS, UMR 9193 - SCALab - Sciences Cognitives et Sciences Affectives, Lille, France; 3 School of Psychology, National Research University Higher School of Economics, Moscow, Russia; 4 Department of Psychology, The University of California, Berkeley, CA USA; 5 Donders Institute for Brain, Cognition, and Behavior, Radboud University, Nijmegen, The Netherlands

**Keywords:** Neuroscience, Psychology

## Abstract

Our senses provide us with a rich experience of a detailed visual world, yet the empirical results seem to suggest severe limitations on our ability to perceive and remember. In recent attempts to reconcile the contradiction between what is experienced and what can be reported, it has been argued that the visual world is condensed to a set of summary statistics, explaining both the rich experience and the sparse reports. Here, we show that explicit reports of summary statistics underestimate the richness of ensemble perception. Our observers searched for an odd-one-out target among heterogeneous distractors and their representation of distractor characteristics was tested explicitly or implicitly. Observers could explicitly distinguish distractor sets with different mean and variance, but not differently-shaped probability distributions. In contrast, the implicit assessment revealed that the visual system encodes the mean, the variance, and even the shape of feature distributions. Furthermore, explicit measures had common noise sources that distinguished them from implicit measures. This suggests that explicit judgments of stimulus ensembles underestimate the richness of visual representations. We conclude that feature distributions are encoded in rich detail and can guide behavior implicitly, even when the information available for explicit summary judgments is coarse and limited.

## Introduction

The natural visual environment contains numerous statistical regularities that can be quantified as feature probability distributions^1,2^. For example, in any natural scene, colors of adjacent locations are likely to be similar because they belong to the same object^3^. The human visual system can exploit these regularities to optimize perceptual inference. Feature probability distributions in the environment are indeed encoded by the visual system at different time scales and are used to make inferences about incoming sensory signals^4–8^. But how precisely are feature distributions encoded? Is all the detail in the feature ensembles lost and replaced with rough summary descriptions?

Representing information about feature probabilities as “summary statistics” (such as mean or variance) may overcome the severe capacity limits of visual working memory and attentional limits on perception^9,10^. Observers can indeed report measures of central tendency and variability [see^11^, for a review]. For example, they can relatively accurately estimate mean and variance for a set of differently oriented lines or an average facial expression for a set of different faces^12^. Reducing sets of items to ensemble summaries requires similar capacity and attentional effort as representing individual items^10,13^. Such summary statistical representations therefore provide a high-level description of visual features and can not only compress visual information but also reduce noise^14^. Overall, claims have been made that representing visual information as summary statistics determines the richness and limitations of conscious visual experience^15^.

However, optimal behavior requires more than summary statistics because observers need a correct model of the environment: a “generative model” in terms of ideal observer approaches^16^. Such models require knowledge of the shape of a probability distribution (for example, a uniform distribution would lead to a different inference than a Gaussian even if their variances are equal). Observers can discriminate different sets of simple stimuli based on mean and variance, but not based on higher-order statistics such as skewness or kurtosis^17,18^. This would be enough for the tasks where observers only have to deal with simple stimuli, such as a 2AFC detection paradigm, but not for many tasks in the real world, where features might have complex distributions. Does this mean that optimal behavior is unattainable? If the detail within the ensembles is thrown away in the summarizing process this could well be the case. But another possibility is that explicit tests of ensemble perception fail to reveal all the knowledge that observers have.

It is well-known that observers sometimes lack explicit access to information that they can nevertheless use to guide behavior. For example, in visual search, observers often search faster when the spatial arrangement of stimuli is constant, even if they are unaware of the repetitions^19^. Here we show that the limits of explicit reporting apply to ensemble perception as well.

We compared observers’ ability to explicitly report ensemble properties with the implicit use of ensembles to guide behavior in a visual search task. Results from a recently developed paradigm, coined feature distribution learning (FDL) show that observers encode more details of feature probability distributions than only summary statistics [see^20^ for an overview]. FDL is based on the well-known priming of pop-out effect in visual search^21,22^. Repeating target or distractor features reduces search times while switching the target and distractor feature slows down searches even more than presenting a new feature (role-reversal): Searching for a blue target among red distractors for a few consecutive trials increases search efficiency, but a sudden switch to a red-colored target slows the search more than a switch to a yellow target. In this way, priming of pop-out allows to understand how observers represent targets and distractors.

**Figure 1:**
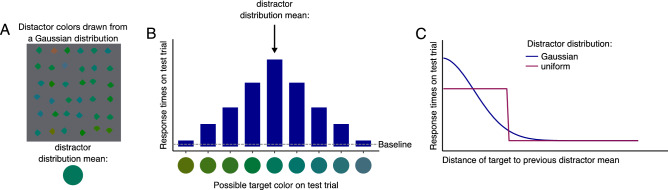
Hypothetical results using Feature distribution learning to assess internal representations of a feature distribution. (**A**) A distractor distribution is presented to the observer in a visual search task. (**B**) Shows a hypothetical response time pattern for different target colors. Response times for targets vary as a function of their probability on the learning trials. (**C**) Presenting many different targets will result in a response function corresponding to the internal representation of the distractors. The shape of that response function resembles the shape of the distractor distribution.

FDL applies this form of attentional suppression of distractor features^23^ to probe the internal model of the distractor distribution. In this paradigm, observers search for the oddly colored target among a large set of heterogeneously colored distractors drawn from a particular distribution^24,25^. During initial learning trials, mean, variance, and shape of the distractor distribution (e.g., whether it is normal, uniform, or skewed) are held constant (Fig. 1A). On subsequent test trials, search times (as a function of the distance in feature space between the preceding distractor mean and the test target) tracked the shape of distractor probability distributions from the learning trials: The target on the test trial is used to probe the encoding of the physical distractor distribution into a probabilistic internal representation. The most probable distractor color (mean of a Gaussian distribution) will lead to the slowest search time and less probable distractor colors to faster search times. The variations in search time on the test trials reflect the attentional suppression. After a set of learning trials, the visual system learns to suppress the distractor features and the degree of attentional suppression depends on the distractor probability. Figure 1B shows a hypothetical response time pattern on test trials for different distractor colors drawn from a Gaussian distribution during learning trials. Presenting many different targets during tests trials will ultimately result in a continuous estimate of the encoded probabilistic representation. The test-trial response times resemble the shape of the physical distractor distribution (Fig. 1C).

Our previous experiments indicated that the result from Feature distribution learning cannot be explained by sampling mechanisms where observers encode only a few items from the distribution^25,26^. Furthermore, observers’ performance with more complex (bimodal) distributions matched predictions of ideal observer models that accurately represent the distribution shape rather than computing only summary statistics^27^ and distribution learning occurred even in the visual periphery^28^. This clearly demonstrated that the visual system encodes feature distribution shape, using it to guide behavior. Internal representations of feature distributions could, in other words, be far more detailed than explicit methods used in ensemble perception have suggested.

**Figure 2:**
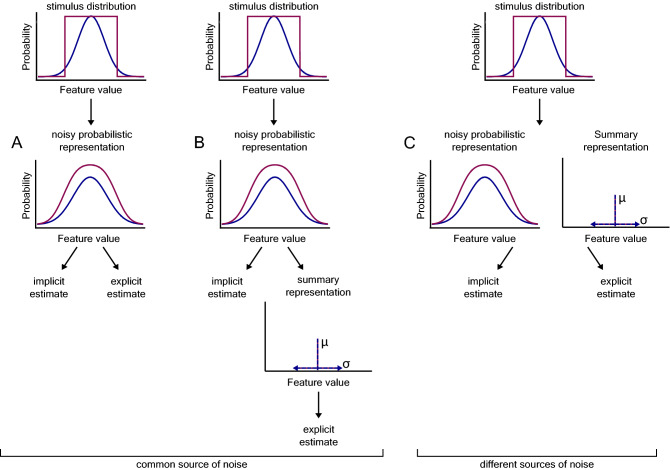
Schematic overview describing potential ways in which the visual system can represent stimulus distributions. A stimulus distribution is encoded as a noisy probabilistic representation (represented here as the stimulus distribution convolved with Gaussian noise) and (**A**) both implicit and explicit estimates are derived from this noisy representation or (**B**) a stimulus distribution is again encoded as a noisy probabilistic representation from which an implicit estimate is obtained and moreover, it allows the estimation of summary representations where the summary statistics of the stimulus representation are encoded, such as range and mean. This summary representation is then used to make explicit estimates about the stimulus ensembles. As a third option (**C**) the stimulus distribution is encoded with two different neural representations: while a noisy probabilistic representation is used for implicit estimates, a separate summary representation encodes the summary statistics of a stimulus distribution. The latter representation enables explicit judgments.

However, it is difficult to directly compare results from this paradigm to studies using explicit reports due to differences in stimuli, task context, and the amount of training. Here, we fill this gap by directly contrasting implicit assessment with the new FDL method and explicit assessment with classic summary statistics methods. Furthermore, we ask if the two approaches measure the same underlying representations. Figure 2 shows an overview of potential ways the visual system may represent stimulus distributions used for explicit judgments and implicit behavior when explicit access is not needed. Figure 2A assumes that both implicit and explicit estimates are calculated from the same noisy probabilistic representation. In Fig. 2B the noisy probabilistic representation is used to directly obtain an implicit estimate of a distribution and a representation of the summary statistics of the distribution that explicit estimates are drawn from. A third, “independent representations”, possibility is shown in Fig. 2C. Here the stimulus distribution is encoded with two different neural representations: a noisy probabilistic representation and a summary representation. Both representations serve distinct purposes: the noisy probabilistic representation is used for implicit estimates and the summary representation includes the summary statistics and is used for explicit judgments. A crucial aspect of this summary representation is that the shape of the two stimulus distributions is not encoded. The summary representation can be a result of an inefficient observer applying a heuristic or sampling just a few items from the set^29–32^. According to this view, the shape of a stimulus distribution is not encoded in the summary representation^11^.

One important characteristic that differentiates the “independent representations” possibility from the other two alternatives is that it does not assume any common noise sources between the summary representation and the probabilistic representation. To take an example, this could occur if observers rely on sampling strategies for explicit tasks but utilize mechanisms for texture or gist perception for implicit tasks [see also ref.^11^]. Here we therefore also asked whether estimates of stimulus variability and measurement precision are correlated between the implicit and explicit tasks. Specifically, we compared the explicit and implicit estimates following the learning of a (truncated) Gaussian distractor distribution.

To preview our results, we found that observers can explicitly estimate the mean and variability of distractor distributions but not their Gaussian shape. In contrast, implicit search time measures showed that the shape of probability distributions was encoded. Furthermore, estimates of variability and measurement precision derived from explicit mean and standard deviation judgments were strongly correlated with one another, but not with estimates from the implicit task. This indicates that explicit judgments share common noise sources that do not affect the implicit task and are therefore unlikely to reflect the same neural representations (depicted in Fig. 2C).

## Results

**Figure 3:**
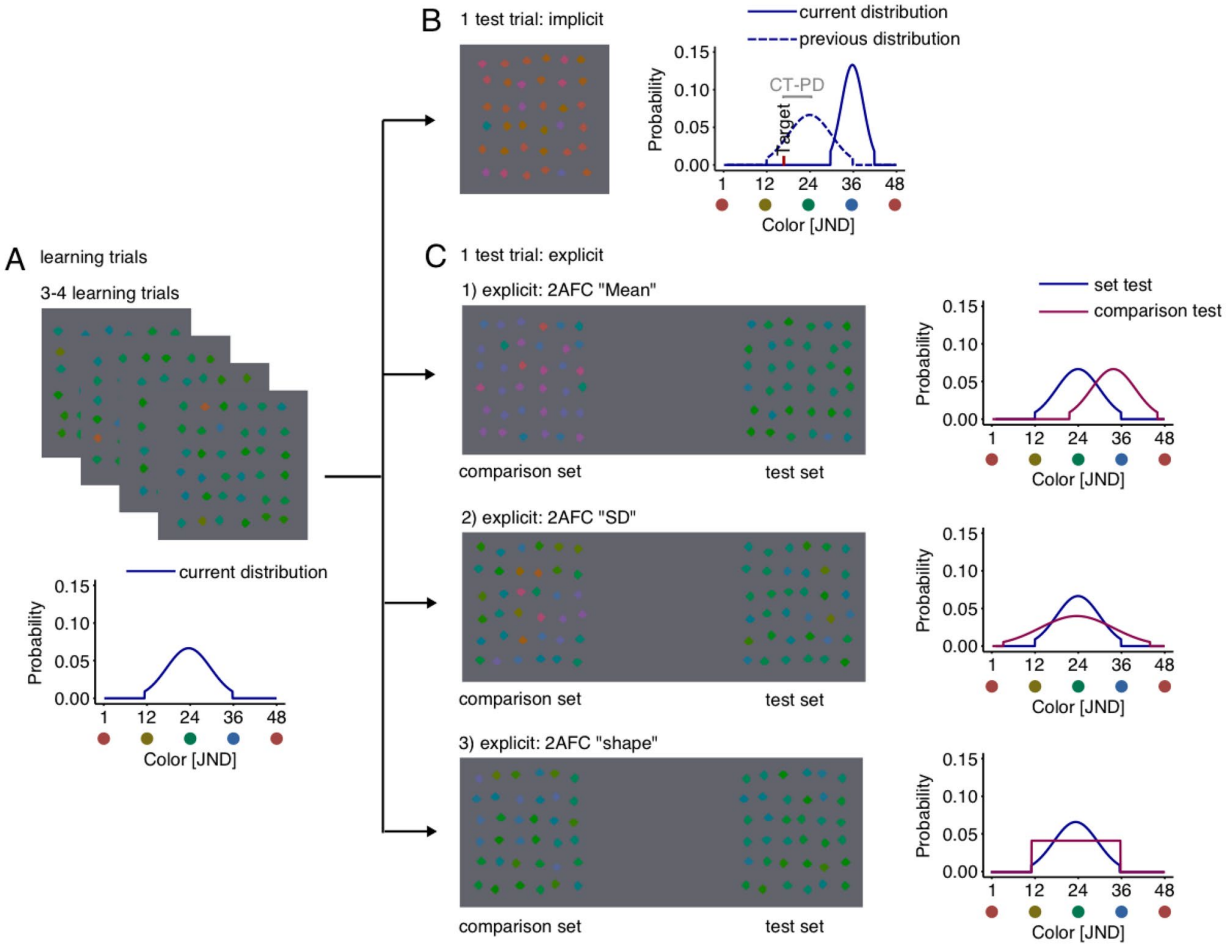
Overview of the experimental procedure. All conditions contained blocks that started with (**A**) learning trials. Learning trials consisted of 3–4 search trials where observers searched for the oddly-colored diamond and reported the location of the cut-off corner. Colors on each learning trial within block were drawn from the same Gaussian distribution but differed between blocks. Implicit learning trials were followed by a test trial (**B**). Here, observers performed another search trial where targets were placed at quasi-randomly chosen probe points around the previous distractor distribution. Explicit learning trials were followed by a (**C**) test trial where observers were presented with two sets of distractors. The colors in the test set were drawn from the same distribution as for the learning trials. The colors of the comparison set were drawn from distributions that differed in mean, SD or distribution shape.

Our observers (n = 18) repeatedly searched for an odd-one-out target (defined by a unique color) over 3–4 consecutive learning trials (Fig. 3A) among heterogeneous distractors with colors drawn from a pre-defined distribution that was kept constant within each block. Their memory of distractor set characteristics was then tested implicitly (Fig. 3B) with response time measures (FDL method) or explicitly, with forced choice tasks (see Fig. 3C).

In the implicit assessment we applied the previously described FDL method. Search times on these test trials are analyzed as a function of the distance in feature space between the current target (that is searched for) and the previously learned distractor distribution mean (hereafter named CT-PD, current target—previous distractors distance). Previous results^20,24–27^ have shown that the RT curve should follow the shape of the encoded distribution, providing evidence that detailed distribution characteristics are encoded.

In the 2AFC tasks, observers were presented with two stimulus sets: a test and a comparison set. The test sets consisted of items drawn from the same distribution as the distractors during learning trials and the distribution used to draw the colors for the comparison set differed in one of the three distribution characteristics: in different sessions, we used comparison stimuli with different means, standard deviations or distribution shapes (see Fig. 5C in Methods). Observers were encouraged to select the set that appeared more similar to the sets from the preceding trials. This method allowed us to explicitly test the encoding of the distractor distribution characteristics.

### Implicit assessment

**Figure 4:**
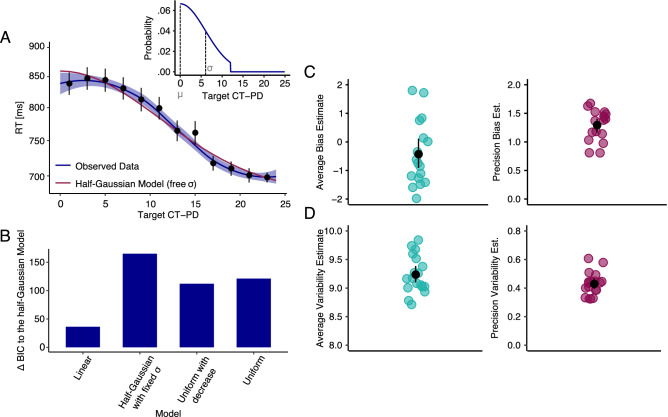
Estimates of ensemble properties obtained with the implicit method. Results plotted in (**A**) show the reaction time as a function of the target to distractor similarity and the best model fit using maximum likelihood estimation. The figure shows the search times on the test trials as a function of the distance between the target on the test trial and the mean of the previous distractor distribution in orientation space. Blue shaded areas show the 95% confidence intervals of the fitted loess function. Observed data after fitting the loess function is plotted in dark blue and the best model (a half-Gaussian model with a free sigma) is plotted in red. Data points correspond to the raw data binned over 2 JND’s across the x-axis. Error bars correspond to the 95% confidence intervals. The small insert corresponds to the underlying distractor distribution. The lower left panel (**B**) shows the differences between the BIC obtained from the best model (half-Gaussian model with a free sigma) and the BIC’s obtained from all other model fits (see text for details on the different models) and (**C**) and (**D**) show the location (μ) and variability (σ) of internal representations. The black data point corresponds to the mean across observers and the error bar represents the 95% confidence interval. Each colored data point refers to one observer.

Overall, observers had a remarkably detailed representation of the distractor distribution, well above the mean and variance only, in stark contrast with typical findings on ensemble perception, but importantly corroborating previous findings of implicit encoding of distractor distributions^20,24–27^. Figure 4A plots the RT curves for the implicit condition and as previously found, the RTs for the current target as a function of the previous distractor distribution (CT-PD) reflect the Gaussian distribution shape. RT’s monotonically followed the distractor probability distribution shape. Search times for targets close to the mean of the previous distractor distribution were slower than search times for targets further away from the mean of the previous distractor distribution. We fitted hierarchical models corresponding to different predefined distribution shapes to the data (with observers as a random factor). We used a uniform model with a fixed range of 12 JND’s, a linear model, and a “uniform with decrease model”, which contains a flat part within the distribution range and a linear decrease outside the distribution range, a half-Gaussian model with SD = 6 and a half-Gaussian model with SD as a free parameter. The best fit was obtained with the half-Gaussian model with a free sigma (BIC =  − 2770.51) with the population-level SD fit = 12.5, followed by the “linear model” (∆BIC = 36.25). Notably, alternative models showed worse fits (uniform: ∆BIC = 121.15; uniform with decrease: ∆BIC = 112.13). A half-Gaussian model with SD equal to the presented distribution (SD = 6) also provided a worse fit (∆BIC = 165.1), suggesting that observers estimates of distractor variability were higher than the actual variability in the stimuli. Figure 4B plots the ∆BIC values from the models and the observed data with the best model fit.

To assess the properties of the internal representation of the encoded distribution we extracted the weighted circular mean (μ, a measure of bias) and SD (σ, a measure of variability) from the data based on the smoothed RT functions. Figure 4C, D plot the average and SD of the extracted circular mean and circular SD for each observer. The left panels correspond to the average bias and variability and the right panels correspond to the SD around these two extracted estimates and serve as a measure of their precision. A precise internal representation of the distractor distribution is centered at zero and has a variability equal to six with SD’s of both estimates (their precision) equal to zero. The mean of the distractor distribution was overall encoded with high precision and the average bias was close to zero, *t*(17) =  − 1.89, *p* > 0.05. The variability of the internal representation of distractor feature distribution was close to 9.2 JND, significantly larger than the variability of the underlying distractor distribution (SD = 6): *t*(17) = 41.86, *p* < 0.001. This matches the model comparison results where the half-Gaussian model with SD = 6 provided a significantly worse fit than a model with free sigma and the free sigma value was estimated to be larger than six. Larger variability for the representation than the true distractor variability is expected because of internal noise.

### Explicit assessment

In the explicit conditions the learning trials were followed by explicit 2AFC judgments of distractors to examine whether the encoded distractor distribution can be accessed using standard ensemble perception measures. These judgments require accessing the encoded feature distributions since the test trial involved explicitly comparing two stimulus sets, selecting the one more similar to the previously seen sets. Observers compared three distribution characteristics: mean, SD and distribution shape (while other characteristics were constant). The test trials consisted of two displays (a test and a comparison set) and observers were asked to select the set that looked more similar to the distractor set on preceding search trials. The test set was always drawn from the same distribution as the distractor distribution during learning trials and the comparison set differed on one of the three distribution features (mean, SD or shape).

**Figure 5:**
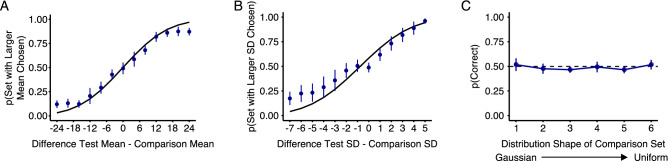
Explicit discrimination of color ensembles differing in (**A**) mean, (**B**) standard deviation, or (**C**) probability distribution shape. In each task, observers have to choose the set that was more similar to the (distractor) ensemble in the preceding visual search trial. In (**A**) and (**B**) the fitted curve corresponds to a cumulative Gaussian using the averaged mean and SD across observers. Data was averaged across all bootstrapped samples and all 18 observers. In panel (**C**) raw data was averaged across all 18 observers. Error bars show 95% confidence intervals.

The data from the three explicit conditions are shown in Fig. 5. Figure 5A,B show the mean and SD comparisons where participants were in both cases able to select the distractor set more similar to the distractor distribution on the learning trials. Importantly, however, the results from the “shape” condition, where observers compared distractor sets that differed in distribution shape, were quite different (Fig. 5C). Observers were clearly unable to explicitly judge which distractor set corresponded to the distractor sets during learning. This contrasts strongly with the results from the implicit FDL method, where differences between uniform and Gaussian distractor distributions were readily distinguished in the CT-PD curves. Performance was even at chance on trials where the colors of the comparison set were drawn from a uniform distribution (rightmost data point in Fig. 5C). Overall this means that while observers have full representations of feature distributions, including their shape, explicit judgments are limited to the mean and SD of the distribution.

### Are implicit and explicit estimates related?

**Figure 6:**
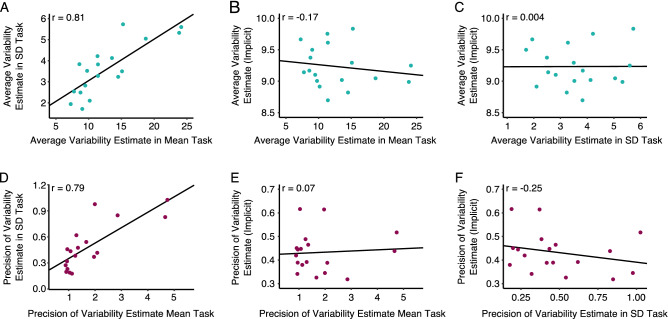
Estimates of uncertainty and precision of uncertainty obtained in the implicit and explicit conditions and their intercorrelations. Correlation between the (**A**) uncertainty estimates from the mean and SD task. (**B**) Correlation between the uncertainty estimates from the implicit and explicit mean tasks. (**C**) Correlation between the uncertainty estimates from the implicit and explicit SD tasks. (**D**) Plots the correlation between the precision of the uncertainty estimates from the mean and SD tasks. (**E**) Plots the correlation between the precision of the uncertainty estimates from the implicit task and the explicit mean task and (**F**) Plots the correlation between the precision of the uncertainty estimates from the implicit task and the explicit SD task. Each data point corresponds to one observer, obtained by averaging the results from the 1000 bootstrapped data sets.

Next, we investigated whether the noise and precision estimates of the implicit and explicit method were related (Fig. 6). A correlation between the estimates of distractor variability representation obtained with different methods would suggest a common noise source and a link between the representations of distractor tapped by these methods in the visual system. Figure 6 plots correlations of the uncertainty estimates obtained from the fitted psychometric functions. Figure 6A plots the average sigma [inverse of the slope/sqrt(2)] obtained from each observer in the SD task against the average sigma from each observer in the mean task. The estimates of uncertainty between the two explicit conditions correlate well: *t*(16) = 5.44, *p* < 0.001, with a Pearson’s correlation coefficient r = 0.81, with Bayes Factor *BF* (A Bayes Factor between 1 and 3 is considered anecdotal evidence for H_1_, values between 3 and 10 provide moderate evidence for H_1_, values between 10 and 30 bring strong evidence for H_1_, values between 30 and 100 bring very strong evidence and values above 100 bring extreme evidence for H_1_. Values between 0.33 and 1 provide anecdotal evidence for H_0_, values between 0.1 and 0.33 provide moderate evidence for H_0_, values between 0.033 and 0.1 provide very strong evidence for H_0_ and values below 0.033 provide very strong and extreme evidence (< 0.01) for H_0_. Descriptions based on Jeffreys^33^ and Lee and Wagenmakers^34^.) = 213.91 indicating strong evidence against the null hypothesis of no correlation. While we found strong positive correlations *within* the corresponding methods, we found no correlation of uncertainty estimates *between* implicit and explicit methods (see Fig. 6B,C), for both the mean and SD tasks: *t*(16) =  − 0.67, *p* = 0.5 and *t*(16) = 0.018, *p* = 0.99 respectively, both with a Pearson’s correlation coefficient of − 0.17 and 0.004, with Bayes Factors *BF* = 0.58 and *BF* = 0.5 which constitutes anecdotal evidence in favor of the null hypothesis of no correlation.

Additionally, we compared the average precision of the uncertainty estimates between the implicit and explicit methods. The motivation for the analysis of the precision of our estimates was the same as for the analysis of the variability (Fig. 6A–C): If the tasks share common sources of noise, then the precision of the estimates should correlate. Figure 6D–F shows correlations between the precision within the explicit methods and between the implicit and explicit method. There was a strong correlation between the precision of the uncertainty estimates of the explicit mean and SD conditions: *t*(16) = 5.1, *p* < 0.001, with a correlation coefficient r = 0.79 and a Bayes Factor *BF* = 129.23 which is strong evidence against the null hypothesis of no correlation. While there were strong positive correlations *within* the corresponding methods, we found no correlation (r = 0.07 and r =  − 0.25) of precision *between* the implicit and explicit methods (see Fig. 6E,F), *t*(16) = 0.27, *p* > 0.05 and *t*(16) =  − 1.04, *p* > 0.05 for the mean and the variance tasks, respectively, with Bayes Factors *BF* = 0.51 and *BF* = 0.73 which constitutes anecdotal evidence in favor of the null hypothesis of no correlation.

In sum, the precision of the implicit encoding does not correlate with the precision of the explicit ensemble estimates. This suggests that the bottleneck in the explicit task does not affect the precision of the implicit estimates.

## Discussion

Our natural environment is full of statistical regularities and therefore contains large amounts of redundant information. During the last decade, several highly influential studies have suggested that the visual system exploits these redundancies in the environment to encode our surroundings as summary statistics of ensembles to save resources and bypass the bottlenecks of attention and working memory. Claims have been made that summary statistics also determine the richness and limitations of conscious experience^15^. But are such simple summary statistics all that is encoded? Or is detailed information about feature distributions retained in some way? Information can be reduced to the mean and variance of a distribution and perceptual tasks like outlier detection or categorization can be performed through knowledge of these statistics^35–37^. However, optimal behavior requires the encoding of a full feature probability distribution that is not always easy to summarize with simple statistics. Explicit reports about summary statistics suggest that neither full feature distributions nor more complex distribution statistics such as skew or kurtosis are encoded by the visual system^17,18^. In contrast, the recently developed feature distribution learning (FDL) paradigm has provided compelling evidence that details of feature probability distributions are encoded through incidental learning, instead of only summary statistics.

We examined the efficiency and precision of visual information encoding for perception and visually guided behavior. While our observers’ explicit judgments about appearance were limited to the mean and the variance of the color distributions, they had detailed representations of feature distributions when measured with FDL methods based on response times. This suggests that explicit judgments about appearance are based on simple heuristics or just a few samples from a full stimulus set that do not allow explicit comparisons of the shape of distractor distributions. Previous results from explicit averaging experiments have suggested that only a small number of stimuli within a set are sampled e.g. the square root of the total items [see ref.^11^ for review] although this view has been challenged^38,39^. Conversely, FDL studies suggest that a large number of items is necessary to obtain detailed internal representations of feature distributions^26^ and a small sample may not suffice to distinguish between distribution shapes.

Our results suggest that explicit summary statistical representations and implicit feature distribution learning are two ways of representing visual information that may follow separate neural paths within the visual system (see Fig. 2C). Supporting this, the precision and uncertainty of the explicit mean and SD estimates correlated with one another, but not with the precision and uncertainty of the implicit estimates. This reveals a common bottleneck in explicit tasks that does not affect implicit estimates and demonstrates a dissociation between implicit and explicit encoding of the characteristics of the distractor distribution. One potential alternative is that the summary representation is based on the probabilistic representation (Fig. 2B) but the noise introduced during the estimating of summary statistics is so large that it masks the correlation between implicit and explicit measures. This is possible given the relatively small between-participant differences in implicit variability measures. Interestingly, this noise may be shared between explicit tasks as demonstrated by strong correlations between mean and SD tasks, potentially indicating that observers suffer from similar limitations in different kinds of explicit reports.

Our results show that detailed information about feature distributions is represented implicitly and can guide behavior. The results also reveal that traditional methods for assessing feature ensemble representations, which rely on explicit judgments of summary statistics, severely underestimate the richness of information available to observers. Some of this information can be revealed only by using implicit methods that rely on observers using as much information as possible to guide behavior. Summary statistics can, nevertheless, be an accurate description of the information available for explicit judgments, and, potentially, for visual awareness. Some studies suggest that explicit judgments of summary statistics might utilize different mechanisms for different features, so the generalizability of the present findings to other features remains to be studied^11^.

Our discovery has profound implications for the understanding of how the visual system stores information and how it is accessed for decision making. Our results provide new insights into the nature of representations of visual ensembles and invite speculation about whether different types of information from encoded feature distributions are available to different visual streams that may serve different functions.

## Materials and methods

### Procedure and stimuli

Search displays consisted of 36 colored diamonds with a single edge cut-off [as in ref.^40^]. Diamonds were presented as a 6 × 6 grid subtending 17.5 × 17.5°.

Thirty-five diamonds were distractors and a single diamond, the target, differed substantially in color. The specific color value for each diamond was drawn from a truncated Gaussian distribution (SD = 6 JND, color values below and above 2 × SD were resampled). The color space was based on 48 isoluminant hues. Adjacent hues were separated by approximately one just-noticeable-difference (JND) obtained for a larger group of observers in previous studies^41,42^. All parameters were based on our previous research with color feature distributions^40,43^.

The experiment consisted of blocks of 3–4 learning trials and a single test trial. On learning trials, participants performed simple searches for odd-color-out targets. They were asked to indicate the position of the edge cut off from the target diamond (up, down, left or right). Following previous studies, we assumed that observers will learn the statistics of distractors during the learning trials (see below for details on target and distractor features).

We included four different conditions for the test trials. In the first “implicit” condition, the test trial was another search task replicating the design introduced in our previous study^20^. In three remaining “explicit” conditions, the test trial consisted of a 2AFC task where observers were presented with two sets of 36 diamonds each and instructed to choose the set that appeared most like the stimulus sets on preceding learning trials. Both sets appeared simultaneously on the screen in counterbalanced positions (left vs. right) across trials. During the search trials observers were instructed to respond as quickly and as accurately as possible and during test trials they were instructed to respond as accurately as possible without being given any feedback. After responding, the next block began.

**Figure 7:**
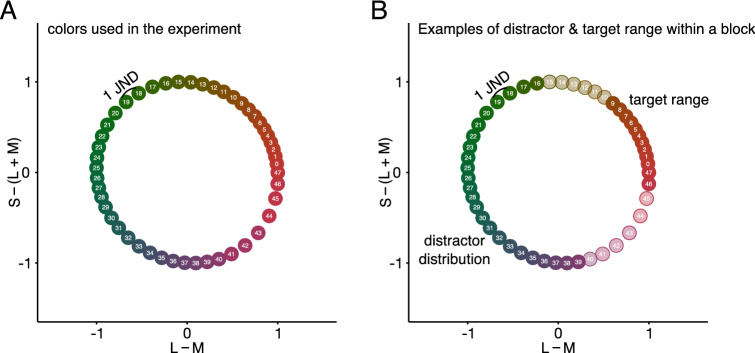
Color wheel with 48 hues used in our study, arranged in DKL space. Neighboring colors are approximately 1 JND apart. The x-axis corresponds to the contrast between L and M cones (L − M), roughly corresponding to the “red-green” dimension. The y-axis corresponds to the variation in S-cone excitation as L + M activation roughly corresponding to the “blue-yellow” dimension. Because of differences in sensory thresholds, colors in some parts of the circle are more distant from each other than in other parts, though differences in JNDs remain the same. (**A**) Full color wheel as used in the study. (**B**) On a particular learning block the distractor distribution and the target color range were constant. Here, the distractor distribution contains a set of greenish colors drawn from a Gaussian distribution with a range of 24 colors. Colors of the targets are obtained from a range that was minimum 18–24 JND away from the mean of the distractor distribution from each side of the distribution. This results in targets being drawn from a range of 12 reddish colors.

In the search task, Gaussian distributions had an SD of 6 JND during learning trials and 3 JND on “implicit” test trials. Outliers with values above or below two SD were removed, so that the distribution range corresponded to 24 JNDs or 12 JNDs respectively. The distractor mean was chosen randomly and kept constant during a learning streak. Target color was chosen randomly during each trial with the restriction that the target-to-distractor-distance was 18–24 JND (see Fig. 7). On implicit “test” trials, the observer’s representation of the previously learned distractor distribution was tested by using targets at different probe points in the feature space around the previous distractor mean. Previous studies show that when a target matches previous distractors, observers are usually slower compared to when it has novel features called the “role-reversal” effect^44^. Many studies have shown that observers respond slower when a test target is similar to previous distractors with the degree of slowing monotonically related to the shape of distractor distributions. This is indeed what we found in our previous studies^24–28,43^. We refer to the test target position in the feature space as the current-target to previous distractor-distance (CT-PD). The distractor distribution means during “implicit” test trials were chosen randomly with the restriction that the target- to distractor distribution mean was at minimum 18–24 JND in either direction along the color wheel. Hereby, we define the distance in an equiluminant and equisaturated color space, so that only the hues change.

In the explicit conditions, test trials consisted of a 2AFC task where the test and comparison display were presented simultaneously. The test display consisted of one set of 36 diamonds with colors drawn from the same distractor distribution as the preceding learning trials and a comparison display where the color distribution differed in one of the three distribution characteristics: mean, standard deviation (SD), or distribution shape while the other characteristics were held constant. In the “mean” and “SD” tasks, colors in both displays were always drawn from a Gaussian distribution. The distance between the mean of the comparison set and the test set in the “mean” task varied from − 24 to 24 in steps of 4. In a second condition (the “SD task”) the SD of the comparison set varied from 1 to 13 JND while the test set had a constant SD of 6 JND. Figure 2 shows a search display as seen during learning trials and three examples of test trials with a test and comparison display. In the last condition (referred to as the 2AFC “shape task”) we varied the shape of the distribution in the comparison set: This display consisted of colors that were drawn from either a Gaussian or a uniform distribution, or a mixture of both distributions using different weights [1 (Gaussian), 0.8, 0.6, 0.4, 0.2 and 0 (uniform)]. Colors of the test set were always drawn from a Gaussian distribution. Mean and SD of the test and comparison set were identical to the mean and SD during learning trials.

### Apparatus

Stimuli were displayed on a 24-inch calibrated LCD monitor (ASUS, VX248h; resolution 1920 × 1080) with Matlab R 2016a and Psychtoolbox-3^45^ that ran on a desktop PC with Windows 10. The screen was color calibrated using a Cambridge Research Systems ColorCal MK2 photometer.

### Observers

Eighteen students or staff from the University of Iceland participated (mean age 30.9 years, 11 female). Students participated for course credits. All (except for two authors) were naïve to the purpose of the study and had all normal or corrected-to-normal vision. Participants with red-green color vision deficiencies were excluded through Ishihara plates^46^ and in a few cases by self-report. Participants gave written informed consent. All experiments were approved by the ethics committee of the National Bioethics committee in Iceland (Vísindasiðanefnd, http://vsn.is) and performed in accordance with their requirements and guidelines and the Declaration of Helsinki. Sample size and trial number were based on the results of previous studies^24–28,43^. In addition, we used model comparison and Bayes Factor analysis to estimate the amount of evidence in favor of our hypotheses rather than more traditional null-hypothesis frequentist testing. Observers that had no previous experience with the search task passed an additional training session containing of 208 blocks of search trials before the first experimental session. Moreover, before each individual session participants underwent an additional training (~ 65 blocks, including either implicit or explicit test trials matching the condition in the session) to familiarize themselves with the task.

The implicit condition and the explicit mean and SD conditions were counterbalanced across 18 participants. For the explicit shape condition, we recruited 7 new participants and 11 old participants (mean age: 29.6, 12 female). All new observers underwent the same training sessions as our previous participants and were also tested on the implicit condition for comparison. Both conditions were counterbalanced across participants.

### Data analysis

All analyses were performed in R^47^. To assess the characteristics of the encoded distractor distribution tested with the implicit procedure we followed procedures described in detail in ref. 20. First, response times (RT) were log transformed and trials with an incorrect response and the trial immediately following an incorrect response were removed from analysis to avoid potential post-error slowing^48,49^.

We then compared the shape of the response time distribution as a function of the absolute current-target to previous distractor-distance (CT-PD) on test trials to the shape of the preceding distractor distribution. This was done by fitting different models to our data with maximum likelihood estimation and comparing the models with the Bayesian information criterion (BIC). The models were selected with methods used in previous studies where observer’s CT-PD functions differed between uniform and Gaussian distributions with the same range^24,25,40,43^. Specifically, following a uniform distribution, observers’ CT-PD functions have a flat segment with low CT-PD (high similarity between target and previous distractor distribution). This reflects the fact that distractors drawn from uniform distributions have a “flat” feature distribution. In contrast, following a Gaussian distribution, the response times monotonically decreased with increasing CT-PD, reflecting monotonically decreasing probabilities of distractors. We expected to observe the same result with the Gaussian distribution used here. Accordingly, our main comparison of interest was a half-Gaussian model against a “uniform with decrease model”, which contains a flat part within the distribution range and a linear decrease outside the distribution range. We also included a uniform model with a fixed range of 12 JND’s (without any decrease) as a control and a half-Gaussian model with fixed SD = 6 to test whether SD’s of distractor representations match physical SD’s. Each model includes a Gaussian-distributed error term (see supplementary information for model equations).

We then obtained the smoothed function of RT’s on test trials depending on the current-target to previous distractor-distance (CT-PD, smoothing was done using local weighted regression, LOESS). This function was then normalized to a discrete probability distribution by subtracting the minima and dividing by the sum of values (the resulting distribution summed to one on a − 90 to 90 degrees range). The mean and standard deviation of this distribution were used as estimates of location and variability of internal representation measured with the implicit method. To reduce the influence of the outliers, this procedure was repeated 1000 times with bootstrap replicates for each observer and then the means (μ) and standard deviations (σ) of each sample were averaged. The standard deviation of each variable (mean and standard deviation) across bootstrapped samples served, in turn, as a measure of the precision of that variable. Note that in contrast to a model-comparison approach outlined above, this is a model-free method that does not make any assumptions about the shape of the CT-PD curve.

For the explicit conditions, we resampled the obtained data creating 1000 bootstrap replicates per observer and fitted cumulative Gaussian psychometric functions (using the ‘quickpsy’ package in R^50^) to each resampled data set and extracted means and SD’s of the underlying Gaussian distribution. The SD of these distributions is an estimate of the underlying noise distribution (SD = inverse of the slope/sqrt(2), mean = PSE). After bootstrapping we calculated the average and SD of both estimates for each observer. The SD of each estimate obtained with bootstrapping corresponds to the precision of the estimate^51^. We then compared these results to those from the implicit FDL method to evaluate whether the uncertainty estimates and precision of these estimates correlate between methods.

Note that the implicit task, explicit mean task, and explicit SD task all provide measures of variability in the representation, but the meaning of these measures is different. For an implicit task, this is an estimate of the variability in distractors (external noise) with the addition of internal noise at different stages of processing. For the explicit mean task, assuming an ideal observer that samples *N* items from the total set of *K* (here, 35 items), the variability estimate is a combination of external noise divided by N and internal noise (potentially shared with the other tasks). Previous studies have suggested that observers use approximately *√K* samples^11^. For the explicit SD task, using a Gaussian approximation for the distribution of the sample standard deviation (Note: the sample standard deviation has a scaled χ distribution yet using this distribution in combination with potential additional noise added after the variance is computed from samples (“late” noise) is computationally problematic when only one set size is used. Hence, we used a simple Gaussian approximation. A scaled χ distribution approaches Gaussian as the number of samples increases and is sufficient for the current goals of estimating the total variability), the variability again depends on external noise, the number of samples used (albeit in a more complex way than for the mean estimation), and internal noise. Importantly, the variability estimates are not expected to be equal, but if the internal noise is shared between any of the tasks, they are expected to be correlated.

## Supplementary information

Supplementary Infromation

## Data Availability

All data have been made publicly available via the Open Science Framework and can be accessed at https://osf.io/fw7vz/.
