# Publisher Correction: Dissociating implicit and explicit ensemble representations reveals the limits of visual perception and the richness of behaviour

**DOI:** 10.1038/s41598-021-87309-5

**Published:** 2021-04-19

**Authors:** Sabrina Hansmann-Roth, Árni Kristjánsson, David Whitney, Andrey Chetverikov

**Affiliations:** 1 Icelandic Vision Lab, School of Health Sciences, University of Iceland, Reykjavík, Iceland; 2 Univ. Lille, CNRS, UMR 9193 - SCALab - Sciences Cognitives et Sciences Affectives, Lille, France; 3 School of Psychology, National Research University Higher School of Economics, Moscow, Russia; 4 Department of Psychology, The University of California, Berkeley, CA USA; 5 Donders Institute for Brain, Cognition, and Behavior, Radboud University, Nijmegen, The Netherlands

Correction to: *Scientific Reports* 10.1038/s41598-021-83358-y, published online 16 February 2021

The original version of this Article contained errors.

Affiliation 2 was incorrectly given as “Sabrina Hansmann-Roth Univ. Lille, CNRS, UMR 9193 – SCALab – Sciences Cognitives et Sciences Affectives, Lille, France” rather than “Univ. Lille, CNRS, UMR 9193 – SCALab – Sciences Cognitives et Sciences Affectives, Lille, France”.

Additionally, there were errors in Figure 4 and Figure 7.

In Figure 4A, the shading around the line graph data points was omitted. In addition, in Figure 4C and D, the solid colour fill in the data points was omitted.

In Figure 7B, the shading for numbers 10 to 15 and 40 to 45 on the colour wheel was omitted.

The original Figures 4 and 7 and accompanying legends appear below.

**Figure 4:**
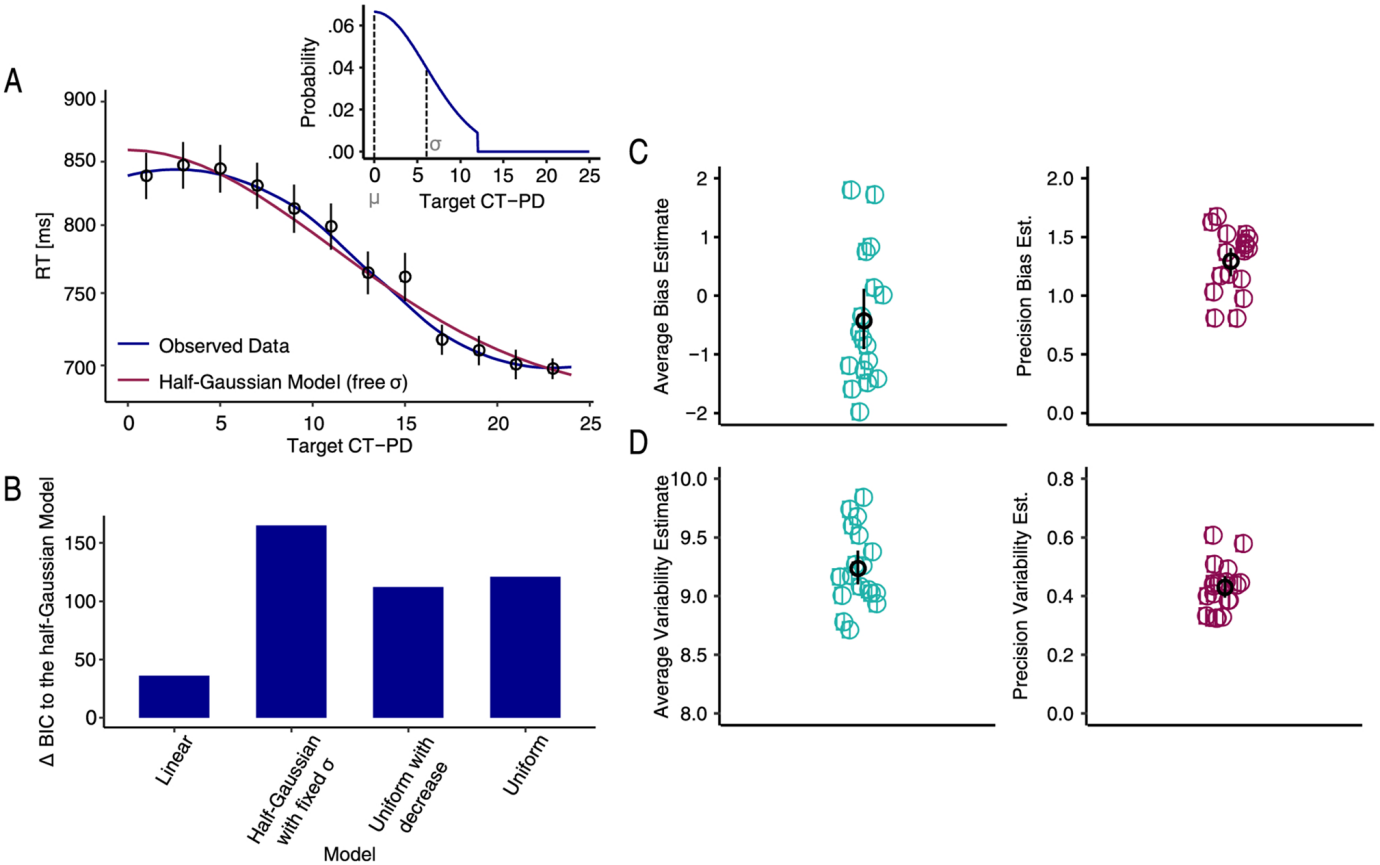
Estimates of ensemble properties obtained with the implicit method. Results plotted in (**A**) show the reaction time as a function of the target to distractor similarity and the best model fit using maximum likelihood estimation. The figure shows the search times on the test trials as a function of the distance between the target on the test trial and the mean of the previous distractor distribution in orientation space. Blue shaded areas show the 95% confidence intervals of the fitted loess function. Observed data after fitting the loess function is plotted in dark blue and the best model (a half-Gaussian model with a free sigma) is plotted in red. Data points correspond to the raw data binned over 2 JND’s across the x-axis. Error bars correspond to the 95% confidence intervals. The small insert corresponds to the underlying distractor distribution. The lower left panel (**B**) shows the differences between the BIC obtained from the best model (half-Gaussian model with a free sigma) and the BIC’s obtained from all other model fits (see text for details on the different models) and (**C**) and (**D**) show the location (μ) and variability (σ) of internal representations. The black data point corresponds to the mean across observers and the error bar represents the 95% confidence interval. Each colored data point refers to one observer.

**Figure 7:**
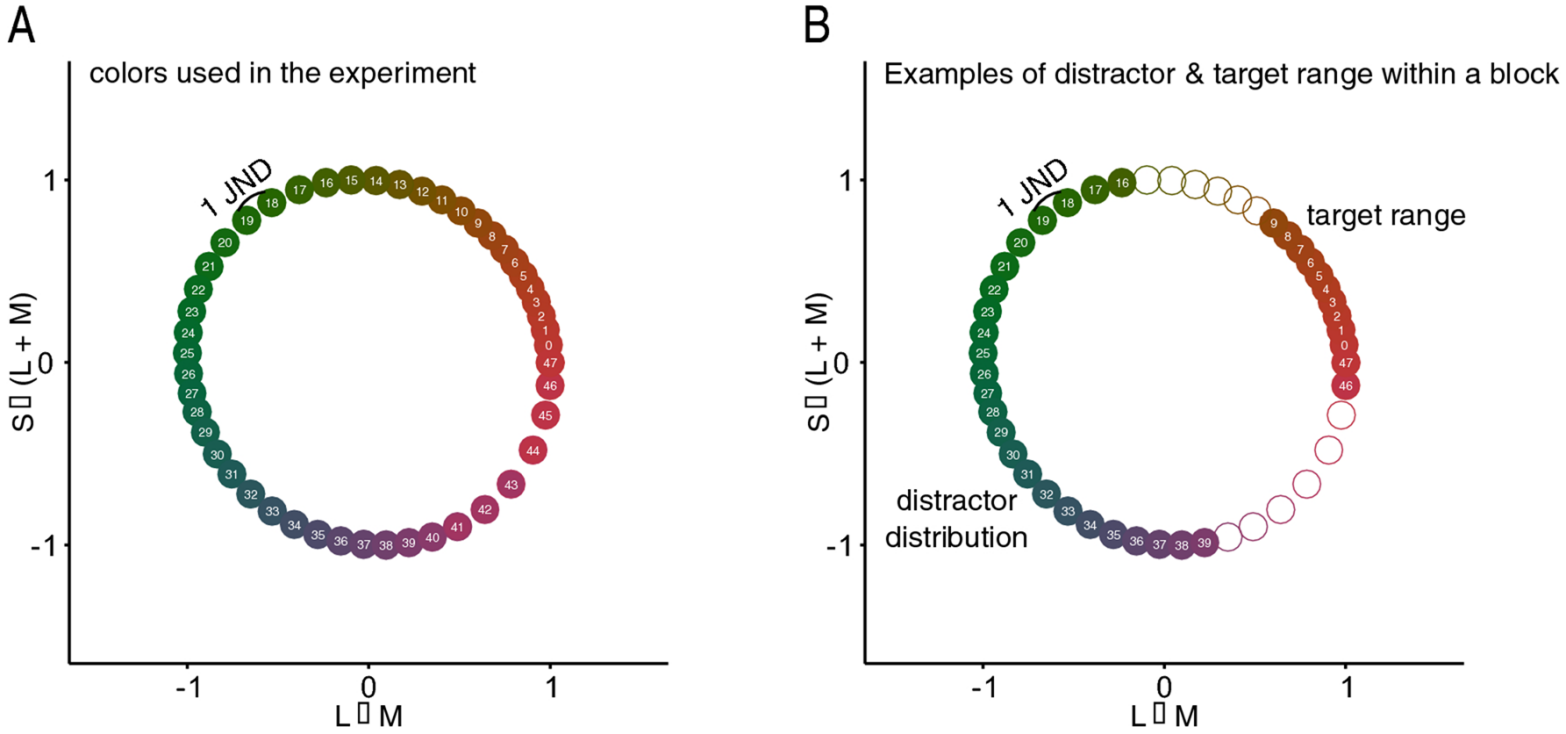
Color wheel with 48 hues used in our study, arranged in DKL space. Neighboring colors are approximately 1 JND apart. The x-axis corresponds to the contrast between L and M cones (L − M), roughly corresponding to the “red-green” dimension. The y-axis corresponds to the variation in S-cone excitation as L + M activation roughly corresponding to the “blue-yellow” dimension. Because of differences in sensory thresholds, colors in some parts of the circle are more distant from each other than in other parts, though differences in JNDs remain the same. (**A**) Full color wheel as used in the study. (**B**) On a particular learning block the distractor distribution and the target color range were constant. Here, the distractor distribution contains a set of greenish colors drawn from a Gaussian distribution with a range of 24 colors. Colors of the targets are obtained from a range that was minimum 18–24 JND away from the mean of the distractor distribution from each side of the distribution. This results in targets being drawn from a range of 12 reddish colors.

The original Article and accompanying Supplementary Information file has been corrected. The corrected Supplementary Information file is also linked to this correction notice.

## Supplementary Information

Supplementary Infromation

