# Supplementary material for: Dissociating implicit and explicit ensemble representations reveals the limits of visual perception and the richness of behavior
Source: Sci Rep. 2021 Feb 16;11:3899. doi: 10.1038/s41598-021-83358-y (PMC7886863; doi:10.1038/s41598-021-83358-y)
Supplement: Supplementary file 1 — Supplementary Infromation [file 41598_2021_83358_MOESM1_ESM.docx]

Supplementary material:

Dissociating implicit and explicit ensemble representations reveals the limits of visual perception and the richness of behavior

Sabrina Hansmann-Roth^1^, Árni Kristjánsson^1,2^, David Whitney^3^ and Andrey Chetverikov^4^

^1^Icelandic Vision Lab, School of Health Sciences, University of Iceland, Reykjavík, Iceland

^2^School of Psychology, National Research University Higher School of Economics, Moscow, Russia

^3^Department of Psychology, The University of California, Berkeley, CA, USA

^4^Donders Institute for Brain, Cognition, and Behavior, Radboud University, Nijmegen, The Netherlands

We fitted pre-defined models of different distribution shapes and the quality of the different fits was assessed with the Bayesian Information Criterion. We have used the following set of models:

Half-Gaussian model with a $\sigma$ = 6:

$RT= c_{0}+2a \times e^{-\frac{{CTPD}^{2}}{2 \times6^{2}}}$,

*where a* defines the height of the peak and $c_{0}$ corresponds to the RT outside the distribution range and the half-Gaussian model with a free $\sigma$:

$$RT= c_{0}+2a \times e^{-\frac{{CTPD}^{2}}{2 \times\sigma^{2}}}$$

Uniform model with a range of $12 JND$:

$$RT= \left\{ \begin{aligned} c_{0}, CTPD\leq12 \\ c_{1}, CTPD>12 \end{aligned} \right.$$

where $c_{0}$, $c_{1}$ determine the RT inside and outside the distribution range.

“Uniform with decrease” model:

$$RT= \left\{ \begin{aligned} c_{0}, CTPD\leq12 \\ c_{0}+b \times CTPD, CTPD>12 \end{aligned} \right.$$

where $c_{0}$ determines the RT within the distribution range and *b* is the decrease outside the distribution range.

Linear model:

$$RT= c_{0}+b \times CTPD.$$

Each model included a Gaussian-distributed error term and random effects corresponding to between-observer variation in model parameters. Different models were fitted to the observed data and the best-fitting parameters were obtained using Maximum Likelihood Estimation.
